# The Case of Surgeon-General Hammond

**Published:** 1864-09

**Authors:** 


### THE CASE OF SURGEON-GENERAL HAMMOND.

Brig.-Gen. William A. Hammond, Surgeon-General of the United States, was tried by a court martial convened by order of the President at Washington on the 15th of January, 1864, consisting of Major-General R. J. Oglesby, United States Volunteers, President; Brig. Gen. W. S. Harney, U. S. A.; Brig.-Gen. W. S. Ketchum, Brig.-Gen. G. S. Greene, Brig.-Gen. W. W. Morris, Col. United States Artillery, Brig.-Gen. A. P. Howe, Brig.-Gen J. P. Slough, Brig.-Gen. H. E. Paine, Brig.-Gen. J. C. Starkweather, with Major John M. Bingham as Judge-Advocate.

The charges are in substance as follows:

*First*—That Surgeon-General Hammond wrongfully and unlawfully, and with an intent to favor private persons in Philadelphia, prohibited Medical Purveyor Cox from purchasing drugs for the army in the city of Baltimore.

*Second*—That he unlawfully, and with intent to aid one Wm. A. Stevens, to defraud the Government of the United States, instructed George E. Cooper, Medical Purveyor in Philadelphia, to buy from Stevens, for the use of the Government, 8,000 blankets of inferior quality which were unfit for hospital use, he, the Surgeon-General, well knowing the blankets were of inferior quality, and that the Medical Purveyor had refused to purchase them.

*Third*—That he corruptly, and with intent to aid Stevens to defraud the Government, gave an order to Stevens to turn over to Medical Purveyor Cooper, at Philadelphia, 800,000 pairs of blankets; whereby he induced the Purveyor to buy on Government account, and at an exorbitant price, 7,677 pairs of blankets, which he had before refused to buy, and for which Mr. Stevens received $3,531,400.*

*Fourth*—That the Surgeon-General, well knowing that Wyeth & Brothers, of Philadelphia, had furnished medical supplies to the Medical Purveyor at Philadelphia, that were inferior in quality, and did corruptly, unlawfully, and with intent to aid Wyeth & Brothers to furnish additional large supplies to the Government, and thereby fraudulently realize large gains, gave the Medical Purveyor an order to have con-

* This is evidently an error. The 800,000 pairs of blankets should be 8000, and the sum paid should be 100 times less than the amount in the text.

stantly on hand hospital supplies of all kinds, for 200,000 men for six months, and directed the Medical Purveyor to purchase a large amount thereof, including $273,000 worth from Wyeth & Brothers.

*Fifth*—That he unlawfully directed Wyeth & Brothers to send 40,000 cans of the extract of beef to various places, and to send the account to the "Surgeon General's office" for payment.

*Sixth*—Conduct unbecoming an officer and a gentleman, in falsely representing that Medical Purveyor Cooper had been relieved from duty at the request of Gen. Halleck.

*Seventh*—That Surgeon General Hammond unlawfully ordered the Medical Storekeeper and Acting Purveyor at Washington to purchase 3,000 pairs of blankets from J. P. Fisher at Washington.

A plea of "not guilty" was entered upon each of the charges and the specifications, and after a full hearing of the testimony for the Government and the defence, and an examination of a large amount of documentary evidence, together with the consideration of the elaborate arguments of both sides, the court rendered a finding of guilty on all the charges, and sentenced the accused to be dismissed the service and to be for ever disqualified from holding any office of honor, profit or trust under the Government of the United States.

Pursuant to the Act of Congress, the record and proceedings were reviewed by Brig. Gen. Holt, Judge-Advocate-General, who delivered an elaborate opinion, concluding as follows:

"That the natural and necessary results of the acts of the accused, as established by the record, involved a criminal spoliation of the Government treasury, which alone would have called for his dismissal from the service, cannot be denied; but when it is remembered, as shown by the proof, that this spoliation was in part accomplished by the purchase of inferior medical supplies and stores, thus compromising the health and comfort and jeopardizing the lives of the sick and wounded soldiers suffering in the hospitals and on the battle-fields of the country—soldiers solemnly committed to the shelter and sympathies of the office held by the accused, by the very law and purpose of its creation, it must be admitted that this fearfully augments the measure of his criminality."

The trial, which lasted nearly four months, was one of the most patient and thorough that has ever occurred in our military history, and the accused had throughout the assistance of eminent and able counsel in conducting his defence.

The court, which was composed of nine General Officers, at the close of this prolonged investigation, declared him guilty of the charges preferred, and awarded the punishment which, in their judgment, was in accordance with the nature of the offences committed, and a careful examination of the record leaves no room for doubt as to the validity of the proceedings, or the justness of the findings and sentence in this case.

The record, proceedings, and sentence of the court in the foregoing case are approved, and it is ordered that Brig. Gen. William A. Hammond, Surgeon-General of the United States Army, be dismissed the service, and be for ever disqualified from holding any office of profit or trust under the Government of the United States. A. Lincoln.

---

Dr. Hammond has added as a sequel to the finding of the Court, the following card:

The undersigned has read in *The Sunday Morning Chronicle* of this city, the remarks of Judge-Advocate-General Holt in the proceedings of the court martial in his case. He learns from this review, and from the order of the President appended, that he has been dismissed the army and prohibited from ever holding office under the United States. The undersigned has no idea that he will lose one friend by this action of the Administration; but his good name is valuable to him, not only as regards those who know him, but those who do not. So soon, therefore, as he is furnished with a copy of the findings and sentence of the court, he will present to the public a brief history of the facts leading to his arrest and trial, a review of the record in his case, and some commentaries on the Report of the Judge-Advocate-General. With these he will be content to submit to the judgment of the world as to how far he has been guilty of the offences charged, and how far he has been the victim of conspiracy, false swearing, and a malignant abuse of official power.

(Signed) William A. Hammond.

Washington, August 22, 1864.

